# Lipogenic signalling modulates prostate cancer cell adhesion and migration via modification of Rho GTPases

**DOI:** 10.1038/s41388-020-1243-2

**Published:** 2020-03-05

**Authors:** Mario De Piano, Valeria Manuelli, Giorgia Zadra, Jonathan Otte, Per-Henrik D. Edqvist, Fredrik Pontén, Salpie Nowinski, Athanasios Niaouris, Anita Grigoriadis, Massimo Loda, Mieke Van Hemelrijck, Claire M. Wells

**Affiliations:** 1 School of Cancer and Pharmaceutical Sciences, Kings College London, London, SE1 1UL UK; 2 Departments of Oncologic Pathology and Pathology, Dana-Farber Cancer Institute and Brigham and Women’s Hospital, Harvard Medical School, Boston, MA 02215 USA; 3 Department of Surgical Sciences, Uppsala University, Uppsala, Sweden; 4 Department of Immunology, Genetics & Pathology, Uppsala University, Uppsala, Sweden

**Keywords:** Cell migration, Metastasis, Cell adhesion

## Abstract

Fatty acid synthase (FASN) is commonly overexpressed in prostate cancer and associated with tumour progression. FASN is responsible for de novo synthesis of the fatty acid palmitate; the building block for protein palmitoylation. Recent work has suggested that alongside its established role in promoting cell proliferation FASN may also promote invasion. We now find depletion of FASN expression increases prostate cancer cell adhesiveness, impairs HGF-mediated cell migration and reduces 3D invasion. These changes in motility suggest that FASN can mediate actin cytoskeletal remodelling; a process known to be downstream of Rho family GTPases. Here, we demonstrate that modulation of FASN expression specifically impacts on the palmitoylation of the atypical GTPase RhoU. Impaired RhoU activity in FASN depleted cells leads to reduced adhesion turnover downstream of paxillin serine phosphorylation, which is rescued by addition of exogenous palmitate. Moreover, canonical Cdc42 expression is dependent on the palmitoylation status of RhoU. Thus we uncover a novel relationship between FASN, RhoU and Cdc42 that directly influences cell migration potential. These results provide compelling evidence that FASN activity directly promotes cell migration and supports FASN as a potential therapeutic target in metastatic prostate cancer.

## Introduction

The 5-year survival rate of patients with localized prostate cancer is ~98% that drops drastically to 28% if the prostate cancer has spread to other parts of the body [1]. Despite the current efforts of early detection, 10–20% of cases present with widespread metastasis at the time of diagnosis [2]. Thus there is an urgent need to understand the molecular drivers of prostate cancer invasion for the development of novel biomarkers of aggressiveness and therapeutics.

A prerequisite of metastasis is the adoption of a migratory phenotype [3]. Cell migration relies on the continuous reorganisation of the actin cytoskeleton, which can be triggered via the activity of Rho family GTPases [4]. Rho GTPases are frequently post-translationally modified by lipids via palmitoylation and/or prenylation, which promotes specific subcellular localization [4]. In addition to changes in motile properties it has long been recognised that cancer cells exhibit alterations in their metabolic activity. This metabolic reprogramming increases the production of metabolic intermediates required to support rapid proliferation [5]. Increased lipogenesis is recognised as a major hallmark for tumour progression with cancer cells switching to dependence on de novo fatty acid (FA) synthesis [5].

The main metabolic enzyme responsible for the generation of FA in the cell is FA Synthase (FASN) [6], which is consistently overexpressed in prostatic tumours [7, 8]. Indeed, changes in FASN expression are an early event in the development of prostate cancer [7] and can be identified as the primary source of palmitate in cancer cells. FASN has been identified as an important enzyme and candidate oncogene as far back as 2009. However, the emphasis has been mainly on FASN activity to promote cellular proliferation [6]. Some more recent studies also suggest that FASN might play a role in promoting cellular migration, where reduced expression of FASN has been associated with impaired cell migration in prostate and colorectal cancer cells [9, 10]. Indeed, FASN was indirectly associated with prostate cancer migration via degradation of the androgen receptor [11]. However, none of these studies focussed on intrinsic migration and the potential downstream pathways that might mediate this response. Importantly metastasis initiating cells upregulate their FA availability via the CD36 receptor [12] and this event is required to drive distal site colonisation. However, the molecular mechanisms through which increased levels of intracellular FA drive metastasis are yet to be elucidated. We therefore sought to understand whether the activity of FASN is essential for prostate cancer cellular migration/invasion and to elucidate the specific molecular pathways that might be impacted when the availability of intracellular palmitate is manipulated through changes in FASN expression levels.

## Results

### FASN depletion suppresses migration and invasion

**Fig. 1:**
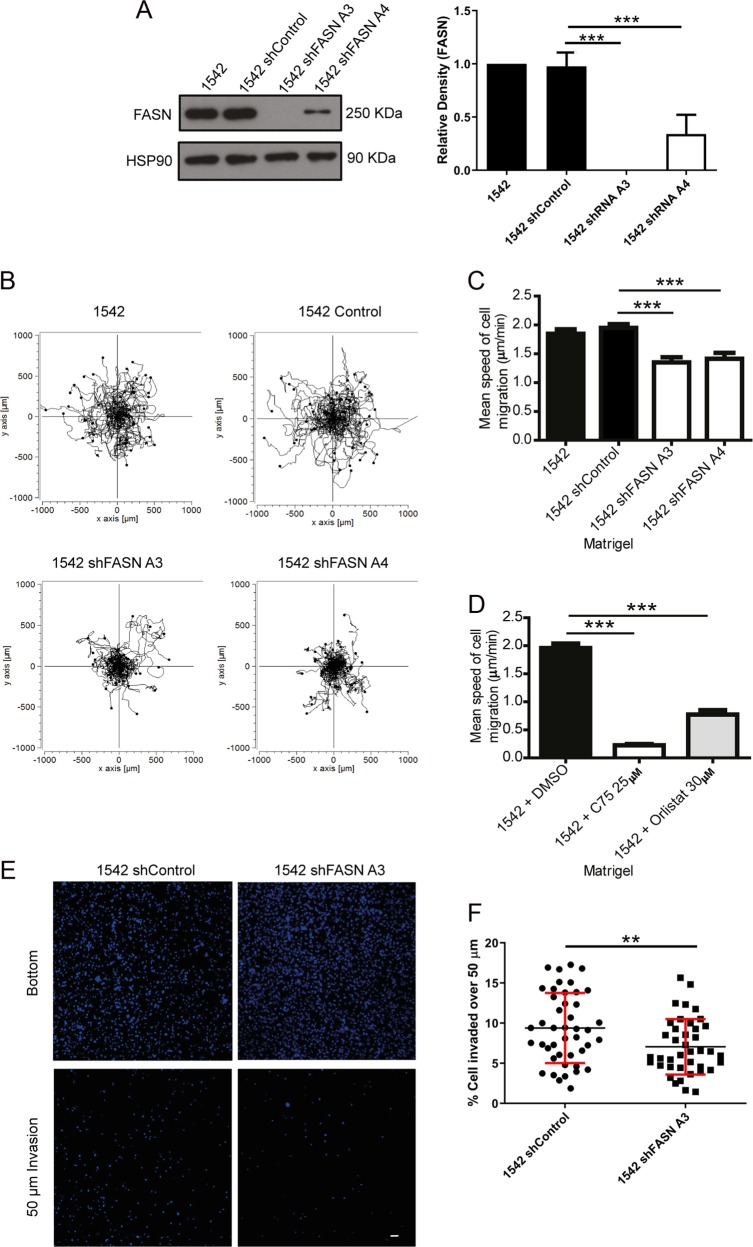
Loss of FASN expression impairs migration and invasion. **a** Cell lysates were immunoblotted for FASN and loading control HSP90. Densitometry analysis was performed and relative quantification of FASN levels calculated. **b** cells were seeded on Matrigel, serum starved, stimulated with 10 ng/ml HGF and imaged for 16 h at 5 min intervals. Migration plots of each cell line (60 cells per condition). **c** Mean (±SEM) speed of migration. **d** 1542 cells were seeded on Matrigel, serum starved, incubated with DMSO, C75 or orlistat and then stimulated with 10 ng/ml HGF. Images were taken and processed as described above (**c**) Mean (±SDev). 60 cells per condition. **e** Cells were overlaid with a type I collagen for 24 h. Cells were fixed and stained with Hoechst. Cells at 0 μm (bottom) and 50 μm were imaged. **f** Percentage of cells invading over 50 μm. All data represent the mean values ± SDev from three independent experiments. Statistical significance was determined by Student’s *t* test. **p < 0.01, ***p < 0.001.

To explore the relationship between FASN and cell migration we reduced FASN expression in PC3 and 1542-CPTX cells (subsequently referred to as 1542 cells) using ShRNAi technology. A total of 1542 cells were derived from a radical prostatectomy and represent cells within the primary tumour with migratory potential [13]. These cells express c-Met (Fig. S1A) and have a migratory response to HGF (Fig. S1B). PC3 cells are an established model of HGF-induced migration [14]. Stable FASN knockdown 1542 and PC3 cells were generated by lentiviral transfection (Fig. 1a and Fig. S1C) using two different shRNA sequences (A3 and A4). Whilst the focus of our investigation was migration we tested cellular proliferation since some migration assays are not suitable for populations with significant differences in proliferation rates. Consistent with previous reports [6] we found a reduced proliferation rate (Fig. S1D) but no evidence for apoptosis (see Movies 1 and 2). We therefore used a direct imaging cell migration assay that is independent of proliferation. We detected a significant reduction in HGF-stimulated mean cell migration in all the FASN shRNA cell lines compared with control (Fig. 1b, c and Fig. S1E). To complement our shRNA study we also utilised pharmacological inhibition of FASN activity, using two well characterised FASN inhibitors (C75 and Orlistat) in our migration assay. In agreement with our shRNA results, inhibition of FASN activity also significantly supressed HGF-induced migration (Fig. 1d, Fig. S1F). It has been previously reported that FASN depletion reduces c-Met expression [9] and thus our data might reflect a loss of HGF signalling in our FASN depleted cells. However although we did detect a modest reduction in c-Met expression, our cells retained a normal biochemical response to HGF (Fig. S2A, B). Furthermore, in a HGF-independent inverted invasion assay FASN shRNA cells were again unable to migrate/invade the collagen matrix when compared with control cells (Fig. 1e, f and Fig. S1G).

### FASN depletion increases cell adhesion

**Fig. 2:**
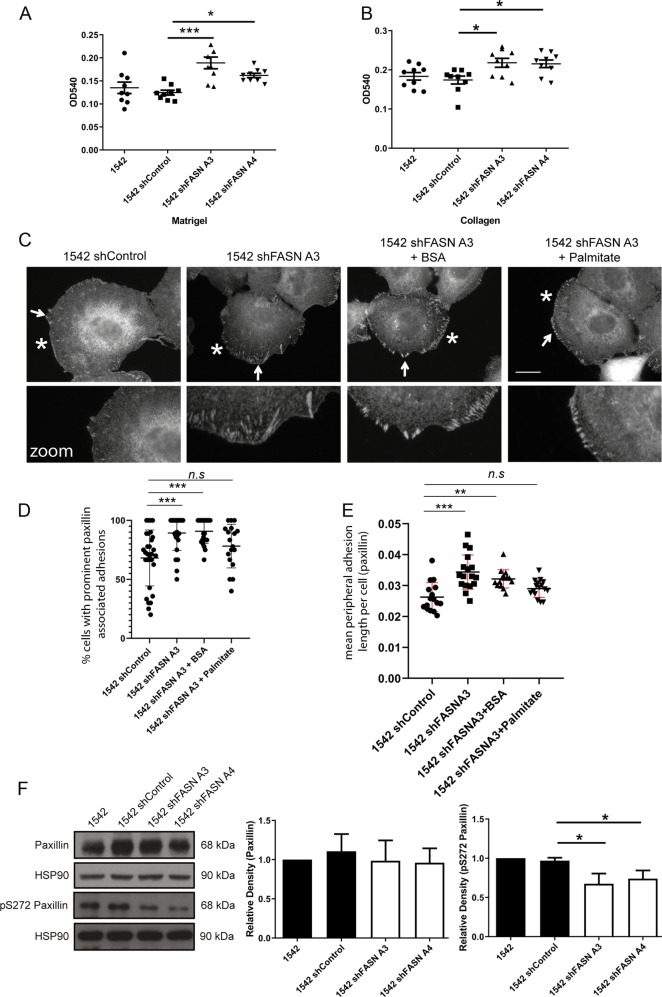
Loss of FASN expression modulates cell adhesion dynamics. Cells were seeded on Matrigel (**a**) or type I collagen for 1 h (**b**). Absorbance measurements at OD540. **c** 1542 shControl, 1542 shFASN A3, BSA-treated 1542 shFASN A3, BSA-palmitate (50 μM) treated 1542 shFASN A3 cells were incubated for 1 h fixed and stained for paxillin. White arrows indicate magnified area. An asterisk (*) indicates a cell with prominent paxillin associated adhesions. **d** Percentage of cells with visible peripheral paxillin positive adhesions. 90 cells counted per condition. **e** Mean adhesion length > 10 adhesions counted per cell. All data represent the mean adhesion length per cell ± SDev accumulated from three independent experiments. Statistical significance was determined by an ANOVA Tukey’s test, *p < 0.05, **p < 0.01, ***p < 0.001, n.s not significant. **f** Cell lysates were probed for paxillin, S272 phospho-paxillin and HSP90 as a loading control. Densitometry analysis was performed and relative quantification of paxillin, S272 phospho-paxillin levels calculated. All data represent the mean values ± SDev from three independent experiments. Statistical significance was determined by Student’s *t* test. *p < 0.05, **p < 0.01. n/s not significant. Bar = 10 μM.

Our data support a critical role for FASN activity during cell migration. A common reason for a reduction in migration speed is an increase in adhesion of cells to the underlying matrix. We therefore tested the adhesion of FASN shRNA 1542 cells to specific matrix. We observed a significant increase in cell adhesion in the FASN depleted population both on Matrigel and collagen I (Fig. 2a, b). Furthermore, detailed analysis revealed that FSAN depleted cells were more likely to display prominent peripheral paxillin associated adhesions (Fig. 2c, d). Similar results were obtained in FASN depleted PC3 cells (Fig. S2C). Indeed, even when control cells exhibited detectable peripheral adhesions (Fig. 2c*) the adhesions in FASN depleted cells were longer (Fig. 2e). Importantly, addition of exogenous palmitate rescued the adhesion phenotype (Fig. 2c–e), whilst having no impact on FASN expressing cells (Fig. S2D) demonstrating the dependence of cells on palmitate to manage optimal matrix adhesion.

Increased adhesion of cell to matrix can either be explained by increased adhesion forming activity or a decrease in the ability of cells to turnover adhesions that are already formed. We have previously shown that the level of paxillin phosphorylation at serine 272 (S272) is a direct measure of adhesion turnover [15], where low paxillin S272 levels are indicative of reduced adhesion turnover leading to more and larger adhesions. We now find that paxillin S272 phosphorylation is significantly reduced in the FASN knockdown cells (Fig. 2f). Furthermore addition of exogenous palmitate raises paxillin S272 phosphorylation in FASN depleted cells back to control levels (Fig. S2E); thus demonstrating a direct link between de novo lipogenesis and cell adhesion turnover.

### FASN depletion reduces RhoU palmitoylation

**Fig. 3:**
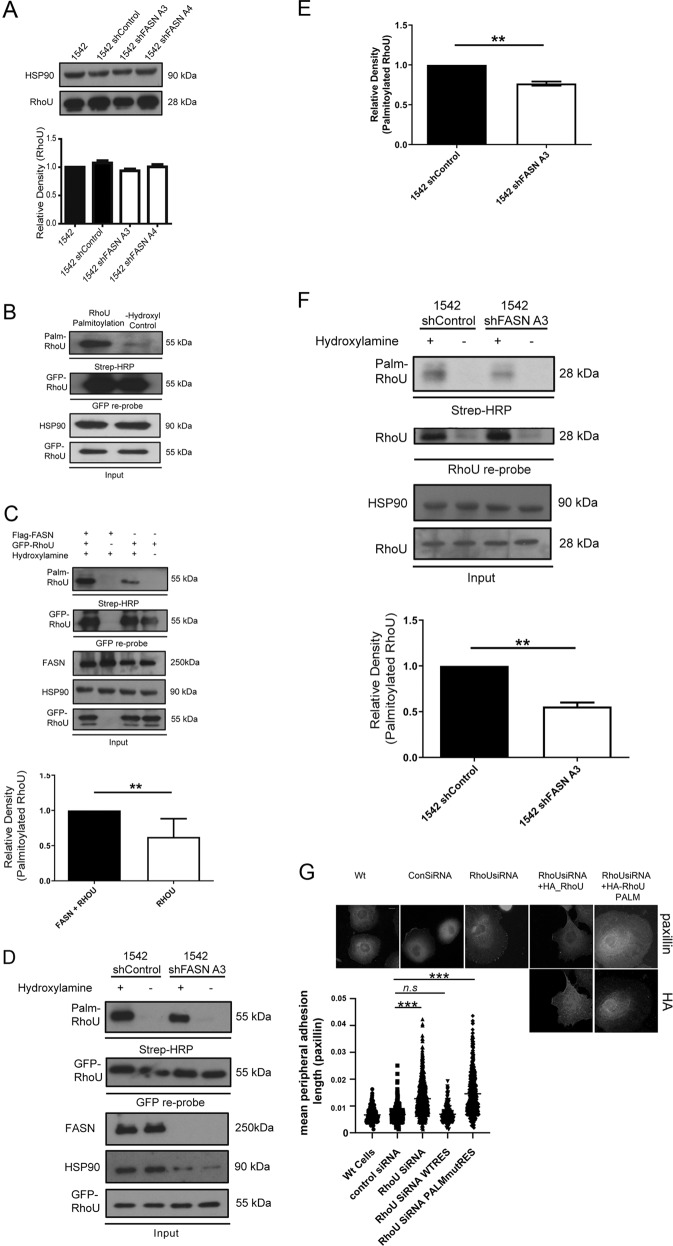
Palmitoylation of RhoU is impaired in a FSAN depleted background. **a** Cell lysates were probed for RhoU and HSP90 as a loading control. **b** HEK293 cells were transfected with GFP-RhoU. Cell lysates were assayed for protein palmitoylation. Biotinylated-BMCC modified samples (palmitoylation positive) were detected using streptavidin-HRP. Omission of hydroxylamine (Hydroxyl) acts as a negative control. Cell lysates were probed with anti-GFP and anti-HSP90. **c** HEK293 cells were transfected with GFP-RhoU in the absence/presence of Flag-FASN, as indicated. Cell lysates were assayed for protein palmitoylation as described above. Cell lysates were probed with anti-FASN, anti-GFP and anti-HSP90. **d** Cells were transfected with GFP-RhoU. Whole cells lysates were assayed for protein palmitoylation as described above. Cell lysates were probed with anti-FASN, anti-GFP and anti-HSP90. **e** Quantification of RhoU palmitoylation in **d**. **f** RhoU was immunoprecipitated using an in-house anti-PAK4 antibody [14]. A palmitoylation assay was conducted on immunoprecipitated protein as described above. In all above densitometry analysis was performed and relative levels calculated. All data represent the mean values ± SDev from three independent experiments. Statistical significance was determined by Student’s t test. *p < 0.05, **p < 0.01. **g** 1542 cells were treated as indicated with control or RhoU siRNA. Cells were then transfected with HA-tagged RhoU-WT or RhoU-PALM (mutant that cannot be palmitoylated). Cells were then fixed and stained for paxillin and expression of HA-tagged protein. Mean adhesion length was calculated and statistical significance determined by an ANOVA Tukey’s test. *p* < 0.001.

Our previous work, in breast cancer, reported that depletion of RhoU expression led to increased peripheral adhesions and reduced paxillin S272 phosphorylation; the same phenotype observed here in FASN knockdown cells [15]. We therefore reasoned that perturbation of RhoU might be directing the FASN phenotype. However, FASN depletion did not change total RhoU protein levels (Fig. 3a). We thus began to consider whether post-translational modification of RhoU was impaired. RhoU is thought to be targeted to membranes by palmitoylation [16] and we confirmed that RhoU can be palmitoylated (Fig. 3b). Given that exogenous palmitate can rescue the adhesion phenotype we hypothesised that perhaps RhoU palmitoylation was under pressure in FASN depleted cells because there was a lack of palmitate. In support of this hypothesis in cells overexpressing both FASN and RhoU we detected an increase in RhoU palmitoylation (Fig. 3c) whilst GFP-RhoU palmitoylation was reduced in FASN knockdown cells (Fig. 3d, e). Most importantly, palmitoylation of endogenous RhoU was also significantly decreased in FASN depleted cells (Fig. 3f and Fig. S3A). These data strongly suggest that FASN activity can influence RhoU palmitoylation. Interestingly we did not detect modulation of Rac palmitoylation (Fig. S3B) nor changes in total Rac levels (Fig. S3C), suggesting that RhoU is particularly sensitive to a reduction in available palmitate in a FASN knockdown background. These data suggest that palmitoylation of RhoU is an important step in adhesion turnover. To confirm this hypothesis, we demonstrated that depletion of RhoU leads to the same changes in adhesion dynamics in prostate cancer cells (Fig. 3g). Then we tested whether a RhoU mutant that cannot be palmitoylated [17] could rescue the RhoU knockdown phenotype. We found that expression of a siRNA resistant RhoU-Wildtype could rescue mean focal adhesion length in cells with depleted RhoU whilst expression of siRNA resistant RhoU-PALM (palmitoylation mutant) could not rescue the adhesion phenotype (Fig. 3g).

### FASN depletion reduces cell spread area via modulation of Cdc42 expression

**Fig. 4:**
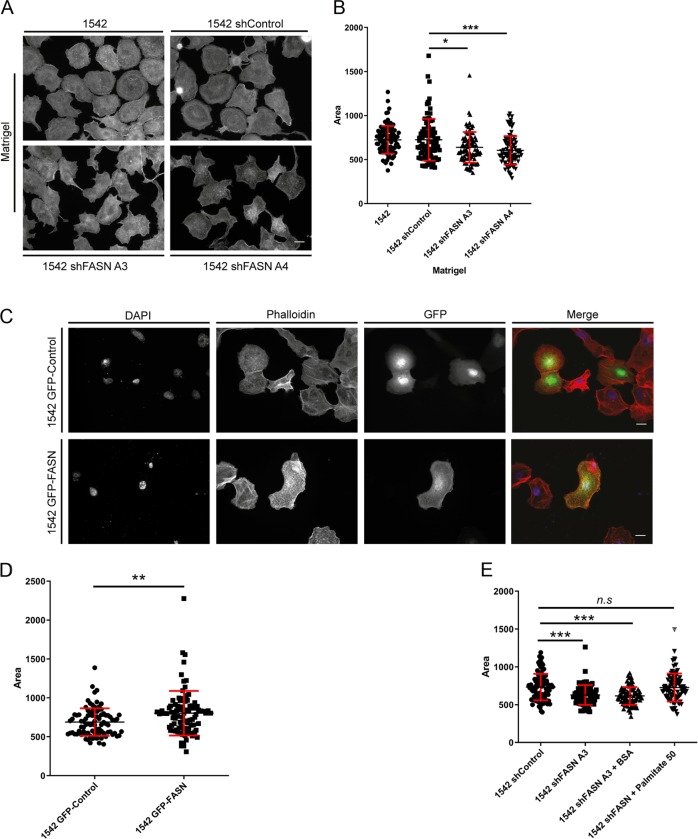
Loss of FASN expression reduces cell spread area. **a** Cells were seeded on Matrigel, fixed and stained for phalloidin. **b** Mean cell area (90 cells per condition). **c** 1542 cells transfected with either GFP alone or GFP-FASN were seeded on Matrigel. Cells were stained with DAPI and phalloidin. **d** Mean cell area (90 cells per condition). **e** Cells were seeded onto Matrigel in the absence or presence of BSA /BSA conjugated-palmitate 50 μM as indicated. Cells were incubated for 1 h fixed and stained for F-actin. Mean cell area (90 cells per condition). In all above data represent the mean values ± SDev from three independent experiments. Statistical significance was determined by an ANOVA Tukey’s test, *p < 0.05, **p < 0.01, ***p < 0.001. n.s not significant. Scale bar = 10 μm.

**Fig. 5:**
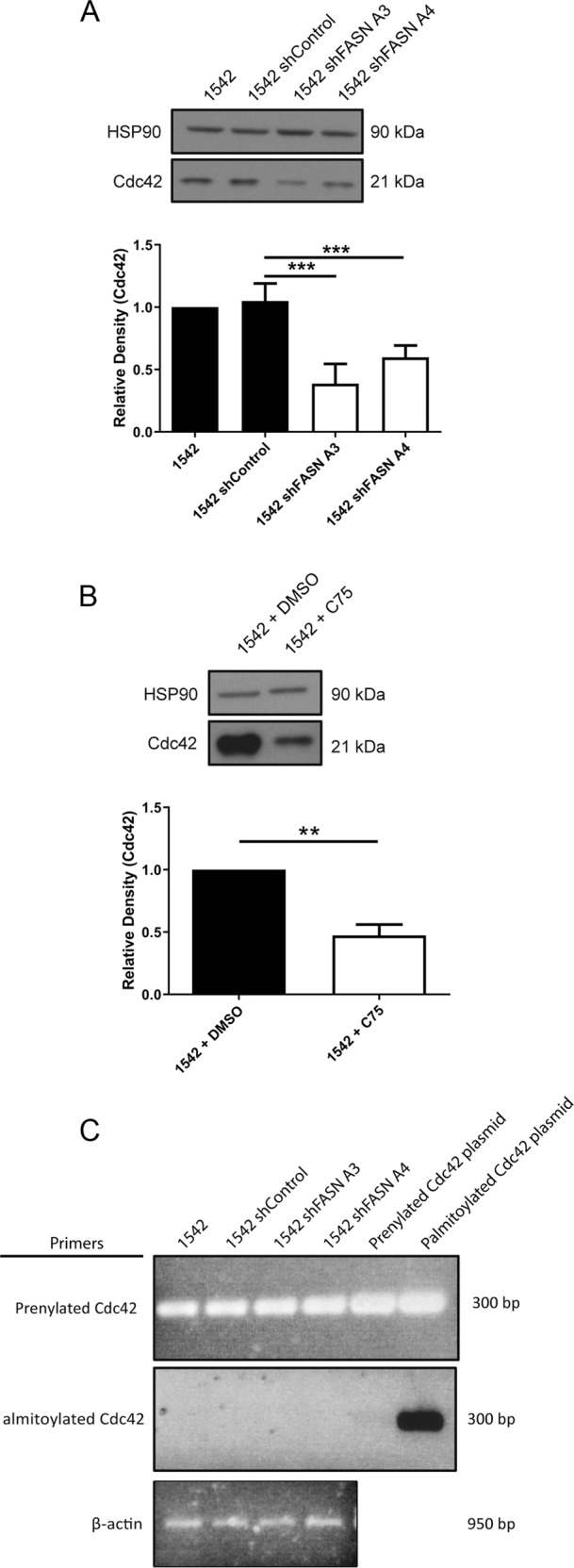
Loss of FASN activity leads to concomitant loss of canonical Cdc42 expression. **a** Cell lysates were probed for Cdc42 and HSP90 as a loading control. Quantification of protein expression was then determined by densitometry analysis. **b** 1542 cells were treated with DMSO or C75 25 μM for 24 h before lysing. Cell lysates were probed for Cdc42 and HSP90 as a loading control. In all above data represent the mean values ± SDev accumulated from three independent experiments. Statistical significance was determined by Student’s *t* test. *p < 0.05, **p < 0.01, ***p < 0.001. **c** cDNA was generated and used in a PCR reaction. PCR primers designed to the prenylated isoform of Cdc42 or the palmitoylated isoform of Cdc42 were used. cDNA plasmids containing either the prenylated or palmitoylated isoform were used as controls. β-actin primers were used as a loading control.

FASN knockdown cells also exhibited a reduction in spread area (Fig. 4a, b and Fig. S4A), whilst overexpression of GFP-FASN increased cell spread area (Fig. 4c, d). Moreover, we again found that addition of exogenous palmitate to FASN depleted cells rescued cell spread area to control levels (Fig. 4e). RhoU has not been linked to cell spread area but another Rho family GTPase Cdc42, which can also be modulated by palmitoylation, has been associated with the regulation of cell spread area [18]. We therefore hypothesised that Cdc42 might be impacted by loss of FASN. In contrast to RhoU we detected a significant loss of Cdc42 protein expression in our FASN depleted cells (Fig. 5a and Fig. S4B). Moreover, we could reproduce the loss of Cdc42 protein levels by treating cells with the FASN activity inhibitor C75 (Fig. 5b and Fig. S4C). Cells can express two variants of Cdc42, one which can only be palmitoylated (referred to here as Cdc42-PALM) or one that is prenylated (referred to here as canonical). We assumed that Cdc42-PALM was expressed by our cells and lost in the FASN depleted cells. However to our surprise, using PCR primers specific to the two variants, we discovered that our prostate cell lines do not generate Cdc42-PALM mRNA rather they exclusively express canonical Cdc42 and the level of canonical Cdc42 mRNA is not reduced by FASN depletion (Fig. 5c and Fig. S4D).

### RhoU regulates canonical Cdc42 protein levels

**Fig. 6:**
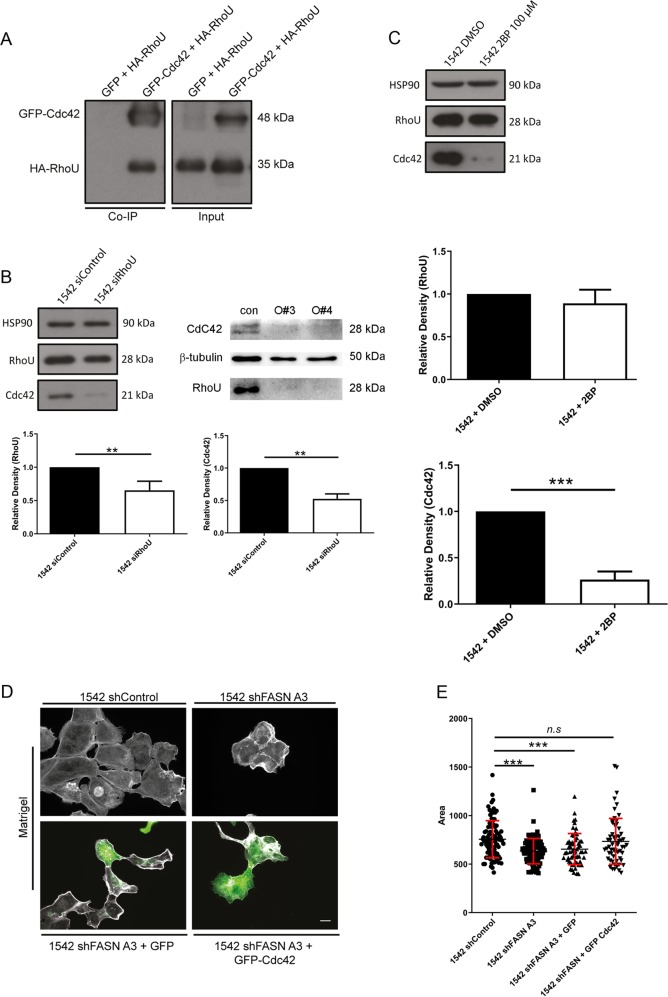
Cdc42 expression is dependent on RhoU. **a** HEK293 cells were co-transfected with either GFP-Cdc42 and HA-RhoU or GFP and HA-RhoU. Cells were lysed and a GFP-TRAP was performed. GFP-TRAP samples and input lysates were probed for GFP and HA simultaneously. **b** 1542 cells were transfected with siControl and siRhoUpool for 48 h (left panel) and 1542 cells were transfected with siControl and siRhoU single oligos (#3 and #4) from pool for 48 h (right panel). Cell lysates were probed for RhoU, Cdc42 and HSP90 as a loading control. Quantification of left hand panel. **c** Palmitate incorporation was inhibited by treating the cells with 2-Bromopalmitate (2BP) 100 μM for 24 h. Cell lysates were probed for RhoU, Cdc42 and HSP90 as a loading control. **d** Cells transfected with GFP or GFP-Cdc42 cells were seeded onto Matrigel. Cells were fixed and stained for F-actin (grey). **e** ImageJ was used to calculate the cell area (60 cells per condition). All above quantification of protein expression was determined by densitometry analysis. Data represent the mean values ± SDev from three independent experiments. Statistical significance was determined by an ANOVA Tukey’s test. *p < 0.05, **p < 0.01, ***p < 0.001. n.s not significant. Bar = 10 μM.

Given that canonical Cdc42 is not palmitoylated we sought to understand how loss of FASN expression could impact on canonical Cdc42 at the protein level. We speculated that perhaps there existed a complex between RhoU and Cdc42 given that other Rho family GTPases interact [19] and found that RhoU and Cdc42 can be immunoprecipitated from cells (Fig. 6a). We then tested Cdc42 expression levels in RhoU depleted cells. Excitingly we detected a dramatic loss of Cdc42 expression in RhoU depleted cells (Fig. 6b). Thus suggesting that Cdc42 protein levels are very sensitive to modest reductions in RhoU expression. Moreover, using a global inhibitor of palmitoyltransferase activity (Fig. 6c) we confirmed that canonical Cdc42 protein levels are dependent on protein palmitoylation. Thus RhoU and Cdc42 are particularly vulnerable to a reduction in intracellular palmitate levels following FASN suppression. Overexpression of canonical Cdc42 in 1542 FASN knockdown cells rescued our cell spread area phenotype (Fig. 6d, e) supporting a specific role for canonical Cdc42.

### FASN, RhoU and Cdc42 expression is increased in high Gleason grade prostate cancer

**Fig. 7:**
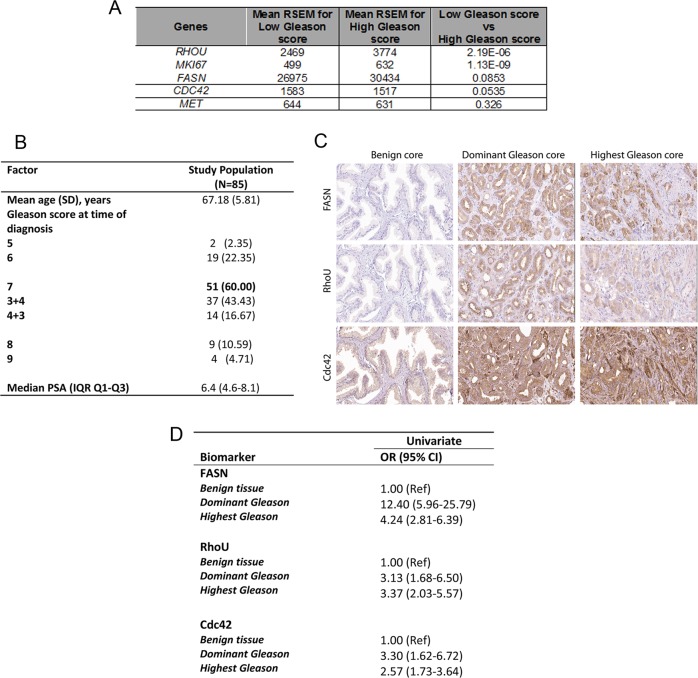
High expression of FASN, RhoU and Cdc42 is detected in human prostate cancer. **a** Normalised RSEM expression levels of MKI67 and RHOU for all samples available in TCGA. The expression of both genes increases with Gleason Score and are significantly different (*t*-test, *p* < 0.00005) when comparing low Gleason score samples (Gleason 6 and Gleason 7 (3 + 4) with high Gleason score (Gleason (4 + 3) to Gleason 10). **b** Baseline characteristics of radical prostatectomy patients included in the U-CAN database. SD standard deviation, IQR interquartile range. **c** Representative immunohistochemistry for FASN, RhoU and Cdc42 in benign tissue and prostatic adenocarcinoma (dominant and highest Gleason): images for each protein were taken from the same core on the same TMA. **d** Univariate odds ratios (OR) with 95% confidence intervals (CI) to predict abnormal expression levels of FASN, RhoU, Cdc42 based on prostate tissue type (i.e. dominant and highest Gleason score tissue versus benign tissue). Movie 1 = Control 1542 cells on Matrigel. Movie 2 = ShRNA A3 1542 cells on Matrigel.

Our results suggest that levels of RhoU and Cdc42 expression in prostate cancer cells are associated and could be correlated with disease progression. Using data held in The Cancer Genome Atlas (TCGA) we found that there is a highly significant difference in RhoU expression and a trend towards significant difference in Cdc42 expression when comparing mRNA signals in low and high Gleason score samples (Fig. 7a). We next evaluated expression in a panel of prostate cancer patient samples from the U-CAN resource [20]. The cohort for this study consisted of 85 men (mean age 67.18) who had undergone a radical prostatectomy. The majority of these men had a Gleason score of 7 (60.00% 51/85), whilst the minority had a Gleason score of 5 (2.35% 2/85) (Fig. 7b). Tissue was stained for FASN, RhoU and Cdc42 expression (Fig. 7c) and the comparative intensity of staining recorded. Univariate analysis revealed that relative to benign tissue, both DG and HG (dominant Gleason-DG and highest Gleason-HG) cancerous tissue types were significantly associated with an increase in the expression of FASN, RhoU, and Cdc42 (Fig. 7d). Taken together this analysis strongly suggests the involvement of RhoU and Cdc42 in prostate cancer progression.

## Discussion

Currently, there is mounting evidence that suggests that FASN is involved in prostate cancer progression, however little is known about the pathways or proteins that act downstream of FASN [21].

This study focussed on investigating whether FASN was involved in regulating proteins that drive cell migration. FASN depletion reduced the migratory capability of prostate cancer cells in both 2D migration and 3D invasion assays. The migration defect was also phenocopied by pharmacological inhibition of FASN activity, thus providing compelling evidence that loss of generation of palmitate rather than loss of non-canonical scaffolding functions are likely responsible for the observed phenotypes. Whilst FASN has previously been reported to be important in the migration of several different cancers [21] this is the first study to focus directly on FASN driven migration and invasion in advanced prostate cancer cell models.

Previous work has identified a relationship between FASN and the androgen receptor [22]. However, the cells studied here represent androgen independent disease and so we sought an alternative explanation for the observed phenotypes. Our exploration of the molecular mechanisms underlying our FASN deletion phenotypes have revealed that a lack of palmitate availability has significant impacts on the ability of some Rho GTPases to function in cells. We discovered that depletion of FASN in prostate cancer leads to a decrease in the palmitoylation of the atypical Rho GTPase RhoU. The silencing of RhoU has been shown to reduce the migratory capacity of PC3 and MDA-MB-231 breast cancer cells [15, 23]. However, nothing is currently known about how reduced palmitoylation affects RhoU-induced cell migration. We propose that a reduction in RhoU palmitoylation leads to a loss of paxillin S272 phosphorylation that is required for adhesion disassembly [15]. This is supported by the observation that addition of exogenous palmitate rescued the adhesion phenotype in FASN knockdown prostate cancer cells. Thus, it could be speculated that the exogenous palmitate taken up by the cells has been specifically utilised to restore RhoU palmitoylation and subsequent paxillin phosphorylation. It is currently unclear how RhoU mediates paxillin phosphorylation, however it has been suggested to be via PKC, PKA (protein kinase A), PKG (protein kinase G) or myosin light chain kinase [15]. Our findings support recent studies demonstrating that cancer cells expressing high levels of the FA receptor CD36 show high metastatic potential [12]. CD36 facilitates the uptake of exogenous palmitate and thus would drive the same pathways we have identified here. Indeed, we would suggest that RhoU activity might be central to the molecular mechanism underlying these in vivo observations [12].

It is interesting that we did not detect impacts on Rac palmitoylation. We would postulate that the majority of Rac is prenylated and so a reduction in palmitate availability is not as impactful. Prenylated Rac may be correctly localised and more readily able to access any residual intracellular pools of palmitate. We anticipate that knockdown of FASN will not lead to a complete loss of intracellular palmitate but rather a significant reduction that is not compensated fully by exogenous palmitate unless we add palmitate in excess. This is supported by our finding that adding excess palmitate to the cell medium rescued the cell behaviour phenotypes.

Whilst Rac was not impacted, this study also identified Cdc42, as directly affected by FASN depletion. FASN knockdown cells were smaller in size and migrated at a reduced speed when compared with their control counterparts. These same phenotypes have previously been reported in Cdc42 knockdown PC3 cells [24]. We detected in the FASN depleted cells a significant decrease in Cdc42 protein levels; with no concomitant reduction in mRNA levels. Interestingly, pharmacological inhibition of FASN also decreased Cdc42 protein expression, suggesting that again lack of palmitate availability was core to the reduction of Cdc42 protein. We speculated that in FASN depleted cells the palmitoylated splice variant of Cdc42 was being affected as a result of reduced palmitate biosynthesis. Indeed, global blockage of palmitoylation also decreased Cdc42 protein levels. However, palmitoylated Cdc42 mRNA was un-detectable in both cell lines. Therefore this data rather suggest that in these prostate cell lines the canonical prenylated isoform of Cdc42 is being affected in response to the loss of FASN activity. In support of this hypothesis canonical Cdc42 re-expression partially rescued the morphological defect seen in FASN knockdown cells. These findings lead us to speculate that perhaps there was a link between RhoU and Cdc42 where modulation of RhoU palmitoylation might account for the destabilisation of Cdc42 protein. Indeed, we found that depletion of RhoU expression lead to a significant reduction in Cdc42 protein expression. Moreover, RhoU and Cdc42 could be co-immunoprecipitated. Whilst not a direct evidence of interaction we would postulate that RhoU and Cdc42 might heterodimerise. It has been shown that several Rho GTPases including Cdc42, RhoA and Rac1 are able to homodimerize [19]; perhaps the considerable sequence homology between RhoU and Cdc42 allows heterodimerisation. Alternatively, these two proteins may be intrinsically linked in a larger protein complex required for Cdc42 stability.

To test the clinical significance of our findings we analysed FASN, RhoU and Cdc42 in prostate cancer and adjacent benign tissue in a cohort of 85 men who underwent a radical prostatectomy. Immunohistochemical staining revealed that the increased expression of FASN, RhoU and Cdc42 was associated with prostate cancer aggressiveness. It is important that both RhoU and FASN expression are increased to deliver an increase in the potential for higher levels of palmitoylated RhoU. Higher levels of palmitoylated RhoU would be predicted to lead to increased levels of Cdc42 stability and thus expression; and this is what we observe in patient tissue. In the absence of increased FASN expression we would predict that despite high RhoU expression, the levels of Cdc42 expression would be unchanged.

In conclusion this study provides evidence that an increased level of FASN expression leads to both a proliferative and invasive advantage. Our results suggest that the invasive advantage is directly related to the increased availability of de novo synthesised palmitate, which supports RhoU palmitoylation mediated cell adhesion turnover and promotes canonical Cdc42 stabilisation. Together these activities help to drive prostate cancer cell invasion. Thus blocking the activity of FASN in prostate cancer cells is an attractive therapeutic pathway.

## Materials and method

### Antibodies and reagents

Rabbit anti-RhoU (Wrch1) from Abcam. Mouse anti-paxillin and anti-FASN from BD Biosciences, rabbit anti-phospho-paxillin S273 (272) from Invitrogen, mouse anti-GFP from Roche, rabbit anti- HSP90 from Santa-Cruz and mouse anti-vinculin from Sigma-Aldrich. HRP-conjugated secondary antibodies from DAKO.

### Cell culture

PC3 prostate cancer cells (ETCC) were grown in RPMI 10% FBS (Sigma-Aldrich) and 100 U/ml penicillin/streptomycin. The 1542-CPTX [13] primary prostate cancer cell line kindly gifted by Prof. John Masters (UCL) was grown in KSFM supplemented with 10% FBS, 0.1 mg/ml bovine pituitary extract, 5 ng/ml EGF and 100 U/ml penicillin/streptomycin. PC3 and 1542 cells were transiently transfected using ViaFect™ (Promega). HEK293 cells (ETCC) were grown in complete DMEM (Sigma-Aldrich) supplemented with 10% FCS (Sigma-Aldrich) and 100 U/ml penicillin/streptomycin and transfected by calcium-phosphate transfection according to the manufacturer’s protocol (Sigma-Aldrich).

### siRNA and shRNA transfection

PLKO.1 lentiviral constructs containing two different shRNA sequences were generated by the RNAi Consortium (Broad Institute Cambridge), see Supplementary methods for all sequences. Supernatant containing lentivirus was used to infect target cells in the presence of 5 μg/ml polybrene. Cells were selected with 2 μg/ml puromycin (Sigma). For transient knockdown cells were transfected with siRNA oligonucleotides using RNAiMax (Thermo Fisher). shFASN A3(CATGGAGCGTAT CTGTGAGAA), shFASNA4 (CGAGAGCACCTTTGATGACAT), shControl (ACAACAGCCACAACGTCTATA). Control siRNA oligo (AATTCTCCGAA CGTGTCACGT) was purchased from Qiagen. The RhoU SMARTpool siRNA oligo (#1- GTACTGCTGTTTCGTATGA, #2- GAACGTCAGTGAGAAATGG, #3- CAGAGAAGATGTCAAAGTC, #4- AAGCAGGACTCCAGATAAA) was purchased from Dharmacon.

### Adhesion assay

Cells were seeded in 96-well plates pre-coated with either 10 μg/ml Matrigel (BD Biosciences) or type I Collagen (Corning). After 60 min at 37 °C, the cells were washed twice with PBS, and incubated in 500 μg/ml methylthiazoletetrazolium (Sigma), and solubilized using DMSO. Absorbance at 540 nm was measured using a plate reader (Perkin Elmer).

### Migration assay

Cells were treated with 20 mM Hepes and 20 ng/ml HGF and placed on a heated (37 °C) stage of an Olympus IX71 microscope. Images were collected using a Retiga SRV CCD camera, every 5 min for 16 h using Image-Pro Plus software. All the acquired time-lapse sequences were displayed as a movie and cells were tracked using ImageJ [25]. Mathematical analysis was carried out using Mathematica 6.0™ notebooks [25], where the mean cell migration speed is calculated for each cell and these data are used to calculate a mean cell migration speed for each population. Statistical significance was calculated using ANOVA accepted for *p* ≤ 0.05.

### Invasion assay

For the inverted invasion assay cells were mixed with type I collagen and then centrifuged to the bottom of a 96-well plate and incubated for 24 h at 37 °C in growth medium. Cells were fixed and stained with formaldehyde: Hoechst. This experiment was performed three separate times. In each experiment 18 wells were seeded with control cells and 18 wells were seeded with ShRNA cells. Any wells damaged during the processing for quantification were discarded from the analysis. The plates were imaged on the A1R confocal microscope where a series of z-stacked images were taken. Thresholding and particle analysis of cells was performed on both planes. The number of cells (as defined by a particles score in Velocity) measured at 50 μm was compared with the number of cells at the bottom of the well (as defined by a particles score in Velocity) to get a relative invasion percentile. Each dot on the graph represents one well and records the % of cells in that well that were able to invade over 50 μm in distance. A mean % invasion percentile was calculated for each condition and a Student’s *t* test was used to calculate the significant difference between the means. Relative % of invasion was calculated by comparing images taken from the bottom of the well against invasion at 50 μm using particle analysis software. See Supplementary data for extended experimental and quantification details.

### Immunofluorescence and image analysis

Cells were seeded onto Matrigel (10 μg/ml; BD biosciences) coated coverslips, fixed in 4% paraformaldehyde and permeabilised with 0.2% Triton X-100. For F-actin staining, cells were incubated with either TRITC- or Alexa fluor 488-conjugated phalloidin (Invitrogen). For detection of paxillin, antibodies were incubated for 2 h at room temperature. Cells were then washed with PBS before incubation with Alexa fluor 647 or 488-conjugated secondary antibodies (Invitrogen) and phalloidin. Stained cells were imaged using either an Olympus IX71 microscope or a Zeiss LSM510 confocal laser-scanning microscope and the accompanying LSM510 software. Focal adhesion number and length were quantified using ImageJ software (NIH). Cells were scored positive for prominent focal adhesions if more than ten paxillin positive adhesions were readily visible at the cell periphery.

### Immunoblotting and immunoprecipitation

Prostate tissue samples (kindly donated by Dr Jonathan Morris) from patients with benign prostatic hyperplasia (G36, G40 and H5) or prostate cancer (F2, F4, D4 and F16) were lysed in RIPA buffer (20 nM Tris-HCl pH 7.4, 150 mM NaCl, 1 mM EDTA, 1% Triton X-100, 0.5% SDS and 1% sodium deoxycholate) and incubated on ice for 20 min. Samples were homogenised with scalpel tearing/vortexing prior to high pulse centrifuging for 3 min at 4 °C followed by additional homogenisation with a needle. The liquid sample was recovered and the appropriate volume of 6 × gel sample buffer added. Samples were then heated at 95 °C for 5 min and stored at −80 °C. Cells were lysed for 10 min in NP-40 lysis buffer [15] and clarified by centrifugation at 13,000 × *g* for 10 min. Proteins were resolved by SDS-PAGE as previously described [15] and immunoblotted with the relevant antibodies. For immunoprecipitation clarified cell lysates were incubated with anti-GFP antibody overnight at 4 °C followed by 1 h incubation with Protein G Sepharose beads (GE Healthcare). The immune complexes were washed and resuspended in 2X SDS loading buffer. Proteins were resolved by SDS-PAGE as previously described [15] and immunoblotted with the relevant antibodies. GFP-TRAP (Clontech) was performed according to the protocol.

### Palmitoylation assay

The protein under investigation was immunoprecipitated from cell lysates. The isolated beads were then incubated with 20–50 mM *N-*Ethylmaleimide (NEM) to block all cysteine residues for 2 h overnight at 4 °C. This incubation Beads were washed to remove excess NEM and then incubated in palmitoylation buffer 1 (1 M hydroxylamine, 50 mM Tris, 150 mM NaCl, 5 mM EDTA, 0.2% Triton X-100, pH 7.4) to remove any palmitate bound to cysteine residues for 2 h at room temperature. Palmitoylation buffer 1 was then removed and substituted with palmitoylation buffer 2 (4 µM BMCC-Biotin, 50 Mm Tris, 150 mM NaCl, 5 mM EDTA, 0.2% Triton x-100, pH 6.2) for 2 h at room temperature. Biotin-BMCC will bind the previously S-acylated protein residues and the resulting lysates can be processed by western blotting. The level of protein palmitoylation is detected with streptavidin-HRP probing for the biotin-BMCC signal. The control for the palmitoylation assay is a duplicate of the lysate that has not been incubated with hydroxylamine. The absence of a signal in the control confirms that in the hydroxylamine positive lysate a biotin-BMCC palmitate switch has occurred. Following incubation beads were thoroughly washed before adding gel sample buffer and storing at −20 °C.

### RT-PCR

Total RNA was isolated from cells using the RNeasy kit (Qiagen). Reverse transcription was carried out with the High Capacity RNA-to-cDNA kit (Applied Biosystems). The cDNA obtained from the RT reaction was then used in a PCR with REDTaq ReadyMix PCR Reaction Mix with MgCl_2_ (Sigma-Aldrich). RT-PCR products were resolved by electrophoresis in 1.5% ethidium bromide-stained agarose gels.

### Immunohistochemistry

Prostate cancer tissues were obtained through the U-CAN project (www.u-can.uu.se) [20]. The tissue microarray (TMA) cohort was constructed from formalin fixed paraffin embedded tumours as previously described [26]. Immunohistochemistry and slide scanning were performed in accordance with standards used at the Human Protein Atlas (www.proteinatlas.org) [27]. Primary antibodies were diluted in UltraAb Diluent (Thermo Fisher Scientific), and applied to the slides for 30 min at room temperature. The slides were further incubated with the secondary reagent (anti-rabbit/mouse horse reddish peroxidase-conjugated UltraVision; Thermo Fisher Scientific) for 30 min at room temperature. Following the washing steps, the slides were developed for 5 min using the avidin–biotin peroxidase staining technique (Vector Elite; Vector Laboratories, Burlingame, CA, USA) using 3,3-diaminobenzidine as the substrate. Slides were counterstained in Mayers hematoxylin (01820, Histolab) for 5 min using the Autostainer XL (Leica), and then rinsed in lithium carbonate water (diluted 1:5 from saturated solution) for 1 min. The slides were dehydrated in graded ethanol and lastly coverlipped (PERTEX, Histolab) using an automated glass coverslipper (CV5030, Leica). The slides were scanned using the automated scanning system Aperio XT (Aperio Technologies). Scoring of TMAs was conducted by pathologists, a specialist registrar in Medical Oncology and a Scientist based at Guy’s and St Thomas’ Hospital, King’s College London and the University of Bologna. FASN, RhoU and Cdc42 staining was dichotomised and assessed as negative/low and high intensity.

### TCGA analysis

Normalised RSEM values from RNAseq for 51 Gleason 6, 171 Gleason 7 (3 + 4), 117 Gleason 7 (4 + 3), 67 Gleason 8, 141 Gleason 9 and 4 Gleason 10 samples obtained from TCGA [28]. Gleason 6 (3 + 3) and 7(3 + 4) patients were combined to form Low Gleason score group and Gleason 7 (4 + 3) − 10 were combined into a high Gleason score group. *T*-test to compare groups was carried out using R3.3 [29].

### Statistical analysis

Data presented is mean ± standard deviation unless stated otherwise. Statistical significance was determined by either ANOVA Tukey’s test or Student’s *t* test. **p < 0.01, ***p < 0.001, where the mean is the average of three independent experiments. All data met the statistical requirements for selected test. Sample size was determined by previous experimental datasets for comparison.

## Supplementary information

Movie_control

movie _shRNA

SFigures
