# Supplementary material for: Lipogenic signalling modulates prostate cancer cell adhesion and migration via modification of Rho GTPases
Source: Oncogene. 2020 Mar 5;39(18):3666–79. doi: 10.1038/s41388-020-1243-2 (PMC7190568; doi:10.1038/s41388-020-1243-2)
Supplement: Supplementary file 3 — SFigures [file 41388_2020_1243_MOESM3_ESM.pdf]

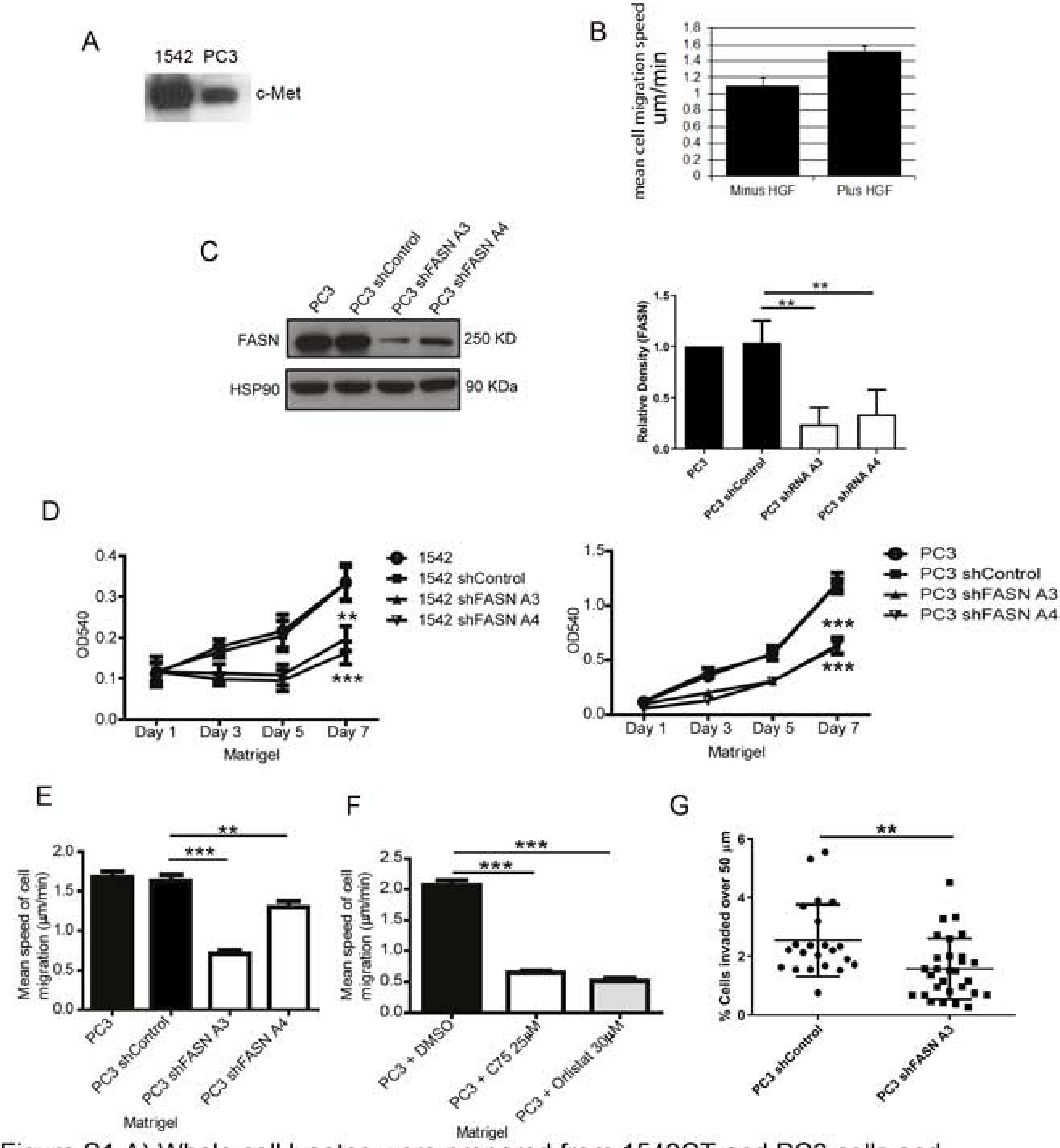

Figure S1 A) Whole cell lysates were prepared from 1542CT and PC3 cells.and immunoblotted for c-Met B) 1542 cells were serum starved, stimulated with 10 ng/ml HGF and imaged for 16 h with images being taken at 5 minute intervals. 30 cells per condition were tracked and the mean migraiton speed calculated. C) Whole cell lysates were prepared from control and FASN knockdown PC3 cells and immunoblotted for FASN and the loading control HSP90. Densitometry analysis was performed and relative quantification of FASN levels calculated.D) indicated cells were left to grow over the course of seven days and subjected to an MTT assay at days 1,-3,-5 and 7. E) and F) control and experimental PC3 cells as indicated were serum starved, stimulated with 10 ng/ml HGF and imaged for 16 h with images being taken at 5 minute intervals. 30 cells per condition were tracked and the mean migraiton speed calculated. G) Control and FASN knockdown PC3 cells were placed in the inverted invasion assay. Data represents the mean values ± SEM (B,D,E&F) +/- SD (C&G) 3 independent experiments. Statistical significance was calcualted using Student's t-test ** $p<0.01$, ***$p<0.001$.

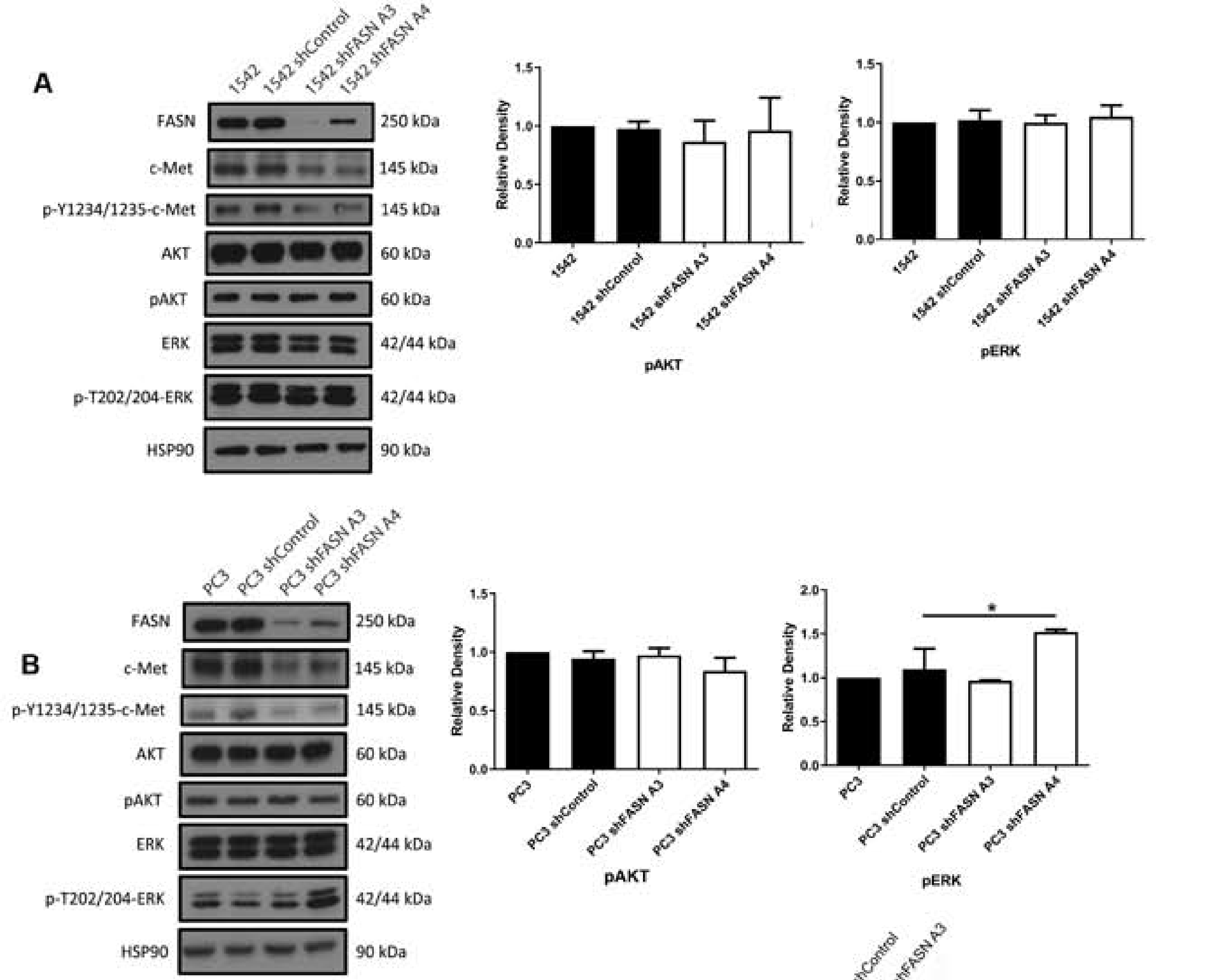

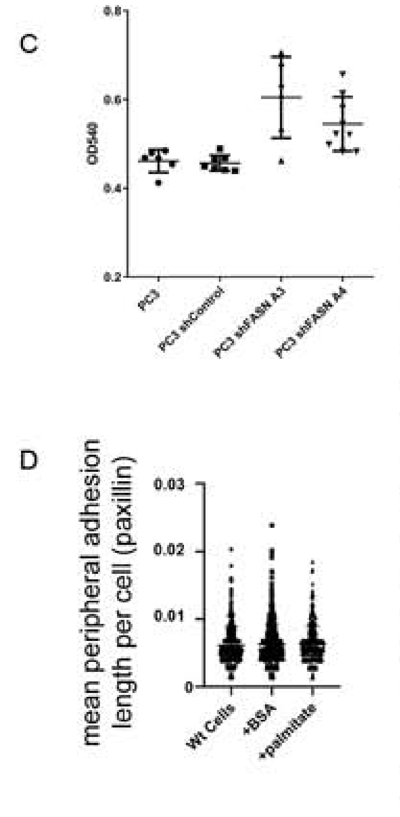

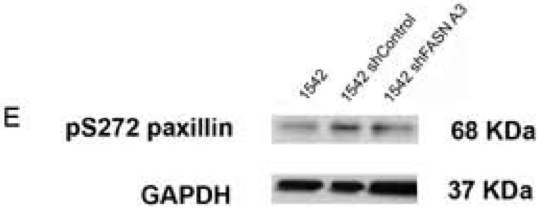

Figure S2 A) and B) Cells as indicated were serum starved for 24 hours before stimulating with 10 ng HGF for 15 mins Lysates were probed for indicated phospho and total proteins with HSP90 as a loading control. Densitometry analysis was performed to monitor pAkt/pERK levels in response to HGF. C) indicated cells were seeded on matrigel. Cells were left to adhere for 1 h before washing, incubation with MTT and subsequent absorbance measurement at OD540.D) 1542 cells were BSA-treated or BSA-Palmitate (50uM) treated incubated for 1 h fixed and stained for paxillin. Mean adhesion length. All data represents the mean adhesion length per cell ± SDev accumulated from three independent experiments E) BSA palmitate was added to 1542 , 1542 control and 1542 ShFASN A3 cells, cells were lysed and probed for pS272 paxillin and GAPDH as a loading control. In all above data represents the mean values +/- SD accumulated from 3 independent experiments. Statistical significance was determined by Students t-test *p<0.05

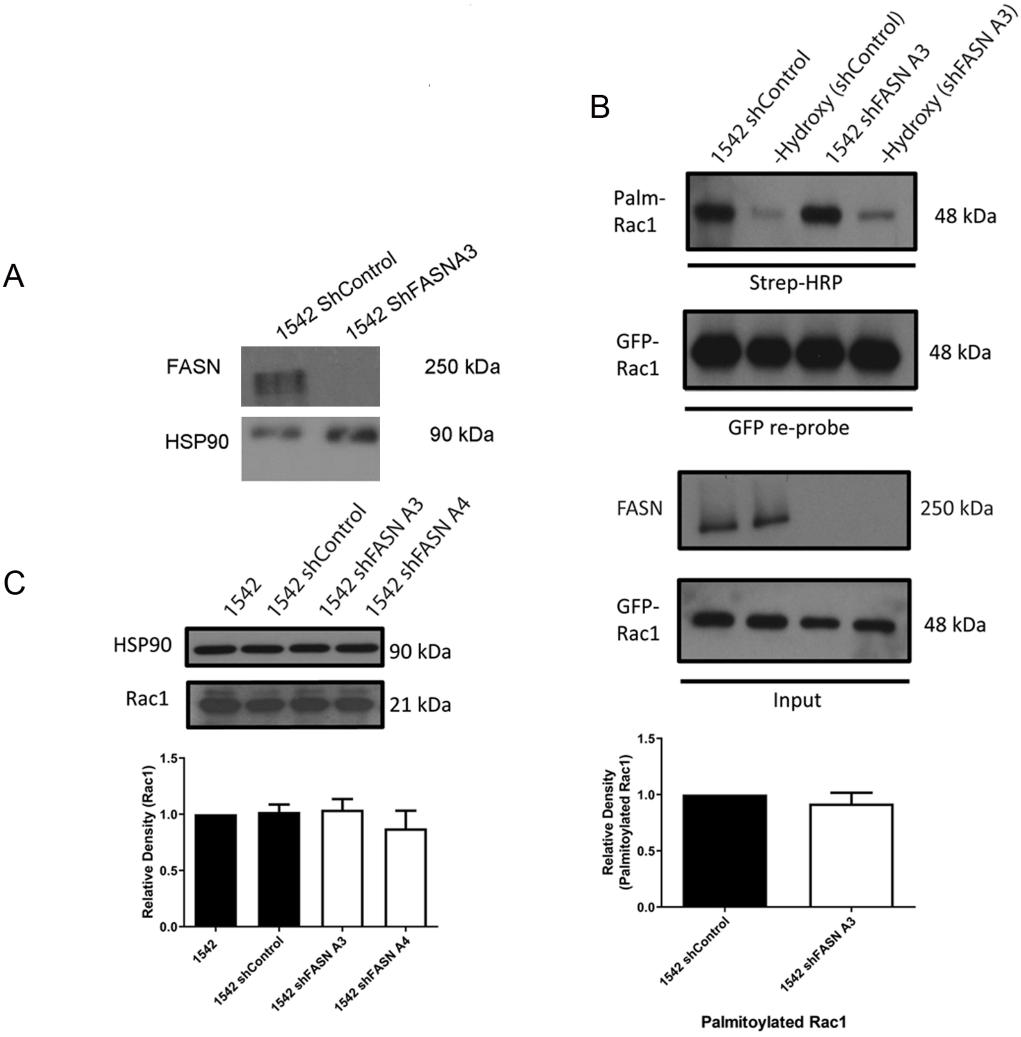

Figure S3. A) Input lysates from Fig. 3F **B)** 1542 shControl and 1542 ShFASN A3 cells were transfected with GFP-RAC1 and incbated for 48 hours. Whole cell lystaes were assayed for protein palmitoylation. Hydroxylamine was used a sa negative control. **C)** Whole cell lysates from indicated cells were probed for total RAC expression. Densitrometry analysis was performed. All data represent mean +/- SDev from 3 independent experiments.

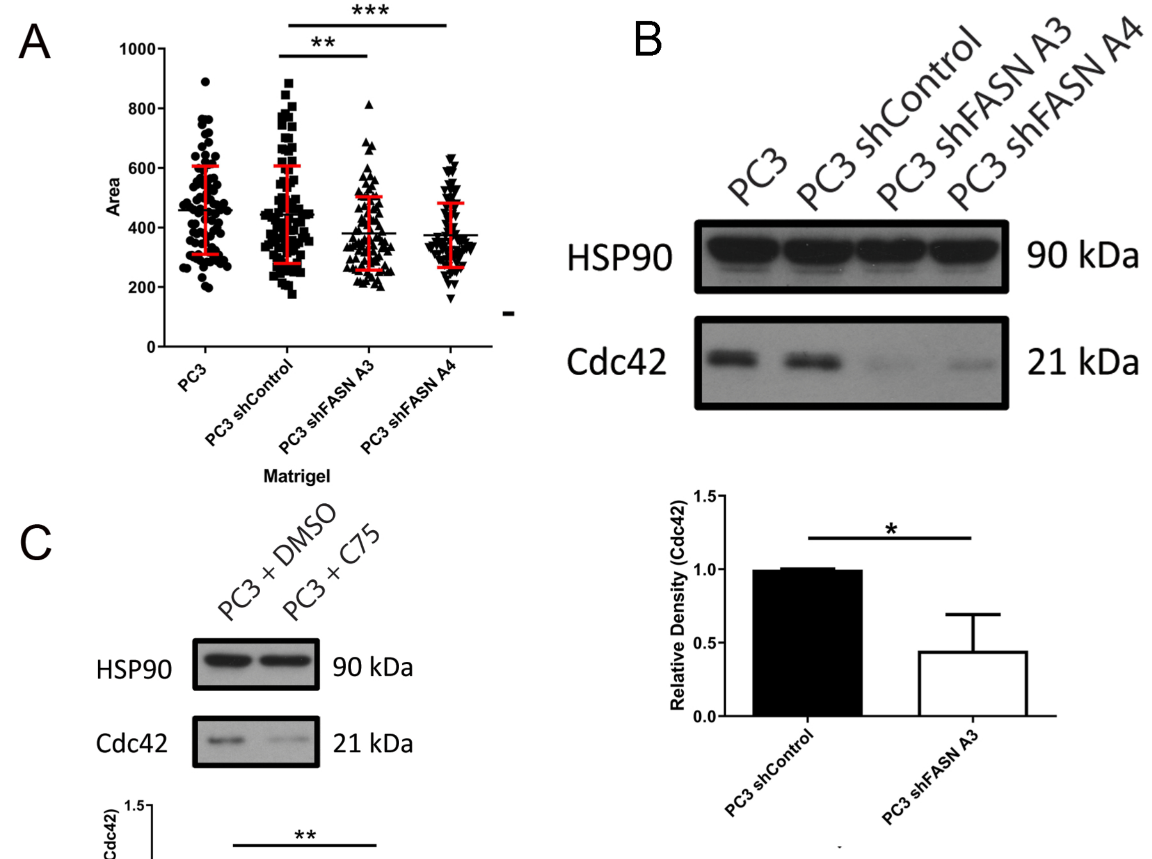

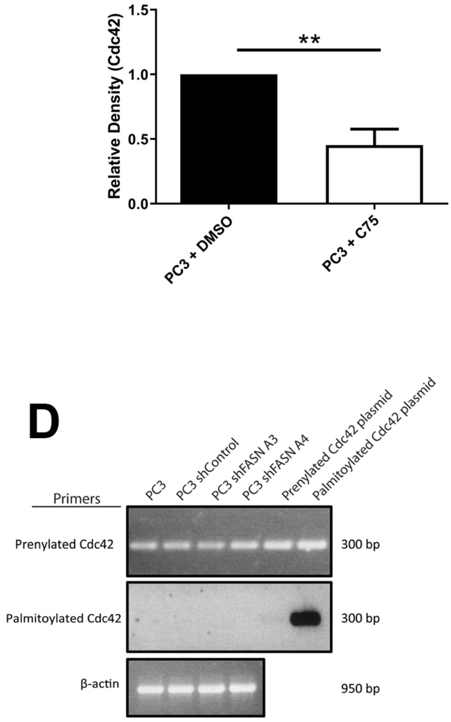

Figure S4. A) Indicated cells were seeded on Matrigel. Cells were fixed and stained for phalloidin. Cell area was calculated (90 cells per condition) B) Indicated cells were lysed and probed for canonical Cdc42 expression. HSP90 was used a loading control. Expression levels were determined by densitometry analysis. C) PC3 cells were treated with DMSO or C75 for 24 h. Lysates were probed for Cdc42 expression HSP90 was used a loading control Expression levels were determined by densitometry analysis. D)cDNA from PC3 cells as indicated was used in a PCR reaction to detect prenylated Cdc42 and palmitoylated Cdc42 signal. cDNA plasmids were used as a positive control with detection of b-actin. All data represents 3 independent experiments mean +/- SDev. Statistical significance was determined by Student's t-test. *p<0.05, **p<0.01, ***p<0.001

Supplemental Methods

si RNA sequences

shFASN A3(CATGGAGCGTATCTGTGAGAA),

shFASNA4 (CGAGAGCACCTTTGATGACAT),

shControl (ACAACAGCCACAACGTCTATA).

Control siRNA oligo (AATTCTCCGAACGTGTCACGT) was purchased from Qiagen.

The RhoU SMARTpool siRNA oligo (#1- GTACTGCTGTTTCGTATGA, #2- GAACGTCAGTGAGAAATGG, #3- CAGAGAAGATGTCAAAGTC, # 4- AAGCAGGACTCCAGATAAA) was purchased from Dharmacon

RhoU expression plasmids

RhoU-HA (Dart et al., 2015) was modified by site directed mutagenesis to yield siRNA resistant RhoU-HA-wt. This construct was then further modified to yield siRNA resistant RhoU-HA-PALM. Modification introduced to change CAAX box to SSFV (Berzat et al., 2005).

Immunohistochemistry

Prostate cancer tissues were obtained through the U-CAN project (19) (www.u-can.uu.se). The tissue microarray (TMA) cohort was constructed from formalin fixed paraffin embedded tumors as previously described (20). Immunohistochemistry and slide scanning were performed at the Swedish SciLifeLab facilities (20). Primary antibodies were diluted in UltraAb Diluent (Thermo Fisher Scientific), and applied to the slides for 30 min at room temperature. The slides were further incubated with the secondary reagent (anti-rabbit/mouse horse reddish peroxidase-conjugated UltraVision; Thermo Fisher Scientific) for 30 min at room temperature. Following the washing steps, the slides were developed for 5 min using the avidin-biotin peroxidase staining technique (Vector Elite; Vector Laboratories, Burlingame, CA, USA) using 3,3-diaminobenzidine as the substrate. Slides were counterstained in Mayers hematoxylin (01820, Histolab) for 5 minutes using the Autostainer XL (Leica), and then rinsed in lithium carbonate water (diluted 1:5 from saturated solution) for 1 minute. The slides were dehydrated in graded ethanol and lastly coverlipped (PERTEX, Histolab) using an automated glass coverslipper (CV5030, Leica). The slides were scanned using the

automated scanning system Aperio XT (Aperio Technologies). Scoring of TMAs was conducted by pathologists, a specialist registrar in Medical Oncology and a Scientist based at Guy's and St Thomas' Hospital, King's College London and the University of Bologna. FASN, RhoU and Cdc42 staining was dichotomised and assessed as negative/low and high intensity.

The Cancer Genome Atlas analysis

Normalised RSEM values from RNAseq for 51 Gleason 6, 171 Gleason 7 (3+4), 117 Gleason 7 (4+3), 67 Gleason 8, 141 Gleason 9 and 4 Gleason 10 samples obtained from The Cancer Genome Atlas (TCGA) (21). Gleason 6 (3+3) and 7(3+4) patients were combined to form Low Gleason score group and Gleason 7 (4+3) – 10 were combined into a High Gleason score group. T-test to compare groups was carried out using R3.3(22).

Quantification of Inverted Invasion Assay:

This experiment was performed three separate times. In each experiment a 96 well plate was used. In each experiment 18 wells were seeded with control cells and 18 wells were seeded with ShRNA cells. Any wells damaged during the processing for quantification were discarded from the analysis. The plates were imaged on the A1R confocal microscope where a series of z-stacked images were taken. Thresholding and particle analysis of cells was performed on both planes. The number of cells (as defined by a particles score in Velocity) measured at 50 micrometres was compared to the number of cells at the bottom of the well (as defined by a particles score in Velocity) to get a relative invasion percentile. Each dot on the graph represents one well and records the % of cells in that well that were able to invade over 50um in distance. A mean % invasion percentile was calculated for each condition and a Students t-test used to calculate the significant difference between the means.
